# Localized Trajectories for 2D and 3D Action Recognition †

**DOI:** 10.3390/s19163503

**Published:** 2019-08-10

**Authors:** Konstantinos Papadopoulos, Girum Demisse, Enjie Ghorbel, Michel Antunes, Djamila Aouada, Björn Ottersten

**Affiliations:** 1 Interdisciplinary Center for Security, Reliability and Trust, University of Luxembourg, L-1855 Luxembourg, Luxembourg; 2 Perceive3D, 3030-199 Coimbra, Portugal

**Keywords:** action recognition, Dense Trajectories, Local Bag-of-Words, spatiotemporal features

## Abstract

The Dense Trajectories concept is one of the most successful approaches in action recognition, suitable for scenarios involving a significant amount of motion. However, due to noise and background motion, many generated trajectories are irrelevant to the actual human activity and can potentially lead to performance degradation. In this paper, we propose Localized Trajectories as an improved version of Dense Trajectories where motion trajectories are clustered around human body joints provided by RGB-D cameras and then encoded by local Bag-of-Words. As a result, the Localized Trajectories concept provides an advanced discriminative representation of actions. Moreover, we generalize Localized Trajectories to 3D by using the depth modality. One of the main advantages of 3D Localized Trajectories is that they describe radial displacements that are perpendicular to the image plane. Extensive experiments and analysis were carried out on five different datasets.

## 1. Introduction

Human action recognition is an active research topic with several applications in surveillance and security [1], healthcare and assisted living [2,3], and human–computer interaction [4]. Nevertheless, due to large differences within the same class of actions, viewpoint variations, occlusions, and changes in lighting conditions, action recognition remains a challenging problem.

Consequently, there is a wide variety of action recognition approaches in the literature. One way to categorize them is based on the area features are computed on: *global* approaches, where the entire image is used to generate features [5,6], and *local* approaches, where specific regions of interest are selected to generate features. One of the most popular approaches belonging to the second category is *Dense Trajectories* [7], in which every action is represented by a set of motion trajectories, along which features are aligned and encoded using the Bag-of-Words (BoW) model [8].

Approaches based on Dense Trajectories are particularly effective when the amount of motion is high [9]. This is mainly because images in a video are densely sampled and tracked for generating the trajectories. However, Dense Trajectories, by definition, include trajectories of points that are irrelevant for action recognition due to background motion and noise, thus resulting in the inclusion of irrelevant information. Furthermore, Dense Trajectories are typically generated using optical flow which fails to describe motion with radial orientation with respect to the image plane. Therefore, taking advantage of the availability of RGB-D cameras, we propose to redefine Dense Trajectories by giving them a local description power. This is achieved by clustering Dense Trajectories around human body joints provided by RGB-D sensors, which we refer to as *Localized Trajectories* henceforth.

The proposed approach offers two main advantages. First, since we only consider trajectories that are localized around human body joints, our approach is more robust to large irrelevant motion estimates. As a consequence, actions which have similar motion patterns, but involving different body parts, are more easily distinguished. Second, our approach allows the description of the relationship of “*action–motion–joint*”, i.e., an action is associated with both; a type of motion and joint location, in contrast to classical Dense Trajectories described by the relationship “*action–motion*” where an action is associated with a type of motion only. This is done by generating features around the Localized Trajectories based on the concept of local BoWs [10]. One codebook is therefore constructed per group of Localized Trajectories. Each codebook corresponds to a specific body joint.

For a better description of radial motion, we further propose to explore Localized Trajectories using the three modalities provided by RGB-D cameras. Specifically, we introduce the *3D Localized Trajectories* concept, which requires the estimation of scene flow, the displacement vector field in 3D, instead of optical flow. Coupling 3D Trajectories and the corresponding motion descriptors with Localized Trajectories offers richer localized motion information, in both lateral and radial directions, allowing better discrimination of actions. However, scene flow estimation is generally noisier resulting in a less accurate temporal tracking of points. Thus, we propose to construct local codebooks by sampling trajectory-aligned features based on confidence and ambiguity metrics [11].

This paper is an extended version of the work in [12]. Compared to our previous work, the main contribution is the generalization of the proposed Localized Trajectories to 3D using RGB-D data. This extension is combined with a novel codebook construction scheme, suitable for tackling noisy feature samples. Moreover, an extensive comparison with state-of-the-art approaches is presented, along with evaluation on multiple datasets and additional discussions and analysis.

In summary, the contributions of this paper are as follows:

1. A novel 2D Localized Trajectories concept is introduced, which utilizes body pose information in order to spatially group similar trajectories together.
2. Localized Trajectories are extended from 2D to 3D thanks to the availability of depth data, which are directly used for 3D motion estimation.
3. A novel feature selection concept for a robust codebook construction is introduced.
4. An extensive experimental evaluation on several RGB-D datasets is presented to validate the discriminative power of the proposed approach.

The remainder of the paper is organized as follows: in Section 2, a literature review of related works is given, followed by a detailed overview of background material in Section 3. The proposed approach is described in Section 4 and Section 5. In Section 6, descriptions of different datasets, experimental setups, and results are presented. Finally, Section 7 concludes the paper and provides a perspective on future research directions.

## 2. Related Work

In this section, we present some of the state-of-the-art action recognition approaches. First, we start by giving a general overview of RGB-D based action recognition approaches. Then, we focus on representations inspired by Dense Trajectories that are directly related to our work.

### 2.1. Dense Trajectories Related Approaches

Initially introduced by Wang et al. [7], Dense Trajectories are classically generated by computing motion and texture features around motion trajectories. Due to their popularity, many researchers have extended this original formulation in order to enhance their performance [9,13,14,15,16].

As a first attempt, Wang et al. [13] proposed to reinforce Dense Trajectories by using the Random Sampling Consensus (RANSAC) algorithm to reduce the noise caused by motion. In addition to that, they replaced the Bag-of-Visual-Words representation with Fisher Vectors.

Then, Koperski et al. [9] suggested enriching motion trajectories using depth information. They proposed a model grouping the videos in two types: videos with a high level of motion and others with a low amount of motion. For the first group, an extension of Trajectory Shape Descriptor [7], which includes depth information has been used, while for the second group a novel descriptor called Speeded Up Robust Features (SURF) has been introduced in order generate local depth patterns.

To further improve the accuracy of recognition, Wang et al. [14] proposed to use deep learned features instead of heuristic spatiotemporal local ones such as Trajectory-Shape Descriptor (TSD) [7], Histogram of Oriented Gradients (HOG) [17], Histogram of Optical Flow (HOF) [18], and Motion Boundary Histogram (MBH) [7].

On the other hand, in [15], a novel approach to encode relations between motion trajectories is presented. Global and local reference points are used to compute Dense Trajectories, offering robustness to camera motion.

Finally, Ni et al. [16] had the idea of focusing on trajectory groups that contribute more importantly to a specific action by defining an optimization problem. Towards the same direction, Jhuang et al. [19] proposed the extraction of features around joint trajectories, increasing the discriminative power of the original Dense Trajectories approach [7].

Although all the aforementioned methods have shown their effectiveness, they unfortunately lack locality information related to the human body. This piece of information is crucial when actions include similar motion patterns performed by different body parts. For this reason, we propose a novel dense trajectory-based approach by taking into consideration the local spatial repartition of motion with respect to the human body.

### 2.2. Action Recognition from RGB-D Data

With the recent availability of affordable RGB-D cameras, a great effort in action recognition using both RGB and depth modalities has been made. For a more comprehensive state-of-the-art, we refer the reader to a recent survey [20], where RGB-D based action recognition methods have been grouped into two distinct categories (according to the nature of the descriptor), namely, *learned representations* [21,22,23] and *hand-crafted representations* [11,24,25]. Since this work deals with the description of actions using Dense Trajectories, we mainly focus on hand-crafted based approaches. In turn, they can be classified as follows: depth-based approaches, skeleton-based approaches, and hybrid approaches.

The first class of methods extracts directly human motion information from depth maps [24,26,27,28,29,30,31,32,33]. The second group gathers approaches which make use of the 3D skeletons extracted from depth maps. During the past few years, a wide range of methods has been designed using this high-level modality [34,35,36,37,38,39,40].

Compared to depth-based descriptors, skeleton-based descriptors require low computational time, are easier to manipulate and can better discriminate local motions. However, they are more sensitive to noise since they widely depend on the quality of the skeleton. Thus, to reinforce action recognition, a third class of methods called *hybrid* makes use of more than two modalities. These approaches usually exploit the skeleton information to compute local features using RGB and/or depth images. These local RGB-D based features have shown noteworthy potential [11,25,41]. Inspired by this relevant concept which aims at computing local depth-based and RGB-based features around specific joints, we propose to adapt the same idea to Dense Trajectories which have been proven to be one of the most powerful action representations.

## 3. Background: Dense Trajectories for Action Recognition

Dense Trajectories were initially introduced by Wang et al. [7]. They are constructed by densely tracking sampled points over an RGB video stream and constructing representative features around the detected trajectories. As mentioned in Section 1, Dense Trajectories have been proven to be very effective in action recognition. They mainly owe their success to the fact that they incorporate low-level motion information. Below, we overview the Dense Trajectories approach.

Let V be a sequence of *N* images. Subsequently, representative points are sampled from each image grid with a constant stepping size—we denote each sampling grid position at frame *t* as $p_{t}=\left(x_{t},y_{t}\right)$. The point $p_{t}$ is then estimated in the next frame using a motion field $\left(u_{t},v_{t}\right)$, derived by the optical flow estimation [42] such that:

(1) $$\begin{array}{c} p_{t+1}=p_{t}+\kappa \cdot \;\left(u_{t},v_{t}\right), \end{array}$$

where κ is a median filter kernel at the position $p_{t+1}$. As a result, large motion changes between subsequent frames are smoothed. Furthermore, to avoid drifting, trajectories longer than the assigned fixed length are rejected. Applying Equation (1) on *L* frames results a smoothed trajectory estimation of the point $p_{t}=\left(x_{t},y_{t}\right)$. We denote the *m*th dense trajectory as:

(2) $$\begin{array}{c} P^{m}=\left\{p_{t_{0}}^{m},\ldots ,p_{t_{0}+L}^{m}\right\}, \end{array}$$

with $\tau =\left[t_{0},t_{0}+L\right]\subset \left[1,N\right]$, m∈{1,…,M}, $t_{0}$ the first frame of the sequence V and *M* the total number of generated trajectories.

The set of *M* trajectories generated in Equation (2) is used to construct descriptors aligned along a spatiotemporal volume. In [7], four types of descriptors are used: TSD [7], HOG [17], HOF [18], and MBH [7]. Each of the above descriptors is designed to capture distinctive spatiotemporal features of the occurring motion. As a final step, all of the descriptors are aggregated and encoded using BoWs—one codebook of visual words per descriptor is constructed using K-means clustering so that the final features are represented by a unified histogram of word appearances.

One of the main drawbacks of Dense Trajectories is that points on the image grid are sampled uniformly, which potentially leads to the inclusion of a significant amount of noise. Furthermore, the generated Dense Trajectories do no take into account the spatial human body structure. Thus, actions with similar motion patterns can potentially be confused during classification.

## 4. Localized Trajectories for Action Recognition

**Figure 1:**
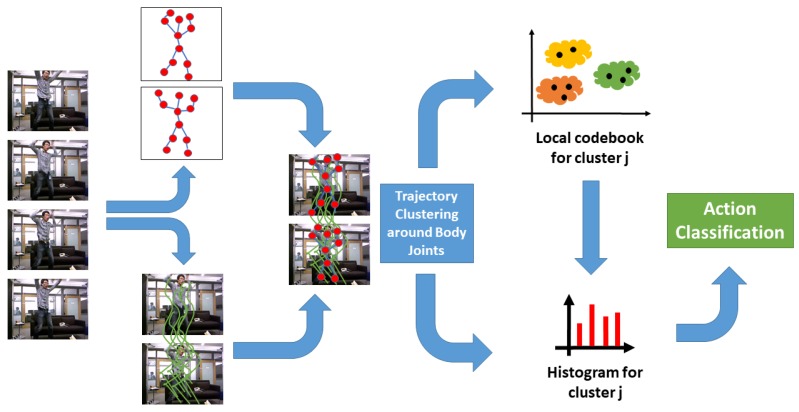
Proposed 2D Localized Trajectories approach. From an RGB sequence, Dense Trajectories are generated and, then, clustered around body joints using RGB-D pose information (only 2D information is used). Finally, local codebooks, for every cluster $G^{j}$, are constructed for the histogram representation of features. This feature representation is used in both training and testing phases of the classification.

To enhance their robustness to irrelevant information, a reformulation of Dense Trajectories is proposed, called Localized Trajectories. The general overview of our approach is illustrated in Figure 1. The main idea of this new approach consists in attributing Dense Trajectories a local description: (1) to track the motion in specific and relevant spatial regions of the human body, more specifically around the joints; and (2) to remove redundant and irrelevant motion information, which can negatively affect the classifier performance.

**Figure 2:**
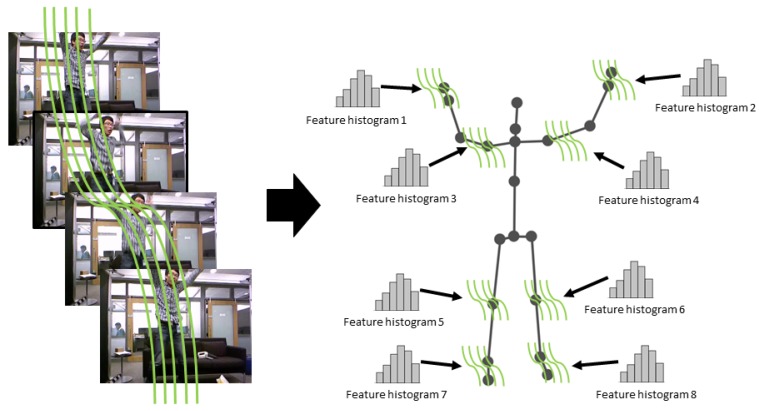
The two stages of Localized Trajectories: Left: clustering motion trajectories around body joints; and Right: local features computation which boosts the discriminative power of the original Dense Trajectories concept.

To that end, the pose information through estimated 3D skeletons is used as prior information to estimate an optimal clustering configuration, as depicted in Figure 2. Let us consider the human skeleton extracted from RGB-D cameras composed of *J* joints and let us denote the trajectory of each skeleton joint *j* as $Q^{j}=\left\{q_{1}^{j},\ldots ,q_{N}^{j}\right\}$. Note that we assume that the joints are always well detected. We use the distance proposed by Raptis et al. [43] to group Dense Trajectories of an action around joints. Given a pair of dense and joint trajectories, respectively, $P^{m}$ and $Q^{j}$, which co-exist in the temporal range τ, the spatiotemporal distance between two given trajectories is expressed using:

(3) $$d\left(P^{m},Q^{j}\right)=\max_{t\in \tau }s_{t}\cdot \frac{1}{L}\sum_{t\in \tau }r_{t},$$

such that $s_{t}=||p_{t}^{m}-q_{t}^{j}{\left|\right|}_{2}$ is the spatial distance and $r_{t}=||\left(p_{t}^{m}-p_{t-1}^{m}\right)-\left(q_{t}^{j}-q_{t-1}^{j}\right){\left|\right|}_{2}$ is the velocity difference between trajectories $P^{m}$ and $Q^{j}$. Then, an affinity matrix is computed between every pair of trajectories $\left(P^{m},Q^{j}\right)$ using Equation (3) as:

(4) $$b\left(P^{m},Q^{j}\right)=\exp\left(-d\left(P^{m},Q^{j}\right)\right),$$

where the measure $d(P^{m},Q^{j})$ penalizes trajectories with significant variation in spatial location and velocity. After a hierarchical clustering procedure which is based on the affinity score [43], a membership indicator function specifies the cluster $G^{j^{*}}$ of joint $j^{*}$ each trajectory belongs to.

(5) $$G^{j^{*}}=\{P^{m},\forall m\in \left\{1,\ldots ,M\right\}\;\mathrm{and}\;\underset{j\in J}{arg min}b\left(P^{m},Q^{j}\right)=j^{*}\}.$$

Furthermore, trajectories that are above a certain threshold of distance are rejected. This condition ensures that irrelevant and noise-resulting trajectories will not be considered, e.g., background motion.

### Feature Representation

As discussed in [7], features can be computed along each trajectory and BoWs can be used to aggregate and encode the information. In such a case, however, a descriptor associated with each trajectory carries no locality information. On the contrary, we propose to exclusively assign trajectories and their corresponding descriptors to trajectory clusters. The main advantage of such a construction is that every trajectory-aligned descriptor does not only capture the spatiotemporal characteristics of the trajectory but it carries its location as well. Thus, we construct a local codebook for each trajectory group $G^{j}$. During feature encoding, one histogram is constructed per joint cluster and per descriptor denoted by $H^{j}$:

(6) $$\begin{array}{c} H^{j}=\left[H_{TSD}^{j}|H_{HOG}^{j}|H_{HOF}^{j}|H_{MBH}^{j}\right]. \end{array}$$

The subscripts of the individual histograms identify the type of descriptors. Finally, an action video is represented by the concatenation of the individual joint histograms in a final histogram *H*, as follows:

(7) $$\begin{array}{c} H=\overset{J}{\underset{j=1}{⋃}}H^{j}. \end{array}$$

## 5. 3D Trajectories and Aligned Descriptors

**Figure 3:**
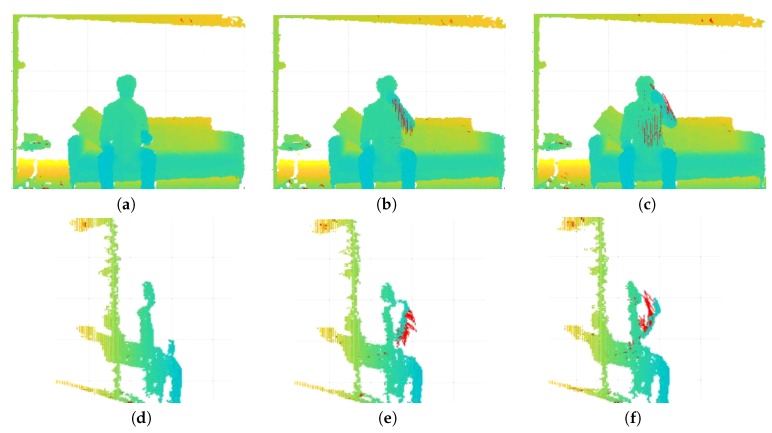
Scene flow-generated motion trajectories. Three phases of the same action are illustrated: (**a**–**c**) the frontal view of a subject drinking water is displayed as a point cloud, along with the corresponding motion trajectories in red; and (**d**–**f**) the same sequence is illustrated from the side. The capture of both lateral and radial motion shape is clearly depicted.

Dense Trajectories, generated via optical flow, offer adequate performance when used for tracking movements that are lateral to the image plane. However, they struggle to track motion that happens radially, due to the fact that the occurring motion is subtle with respect to the 2D image plane. Consequently, in this section, we propose to extend localized Dense Trajectories to RGB-D input video stream by replacing optical flow with scene flow. The generated 3D trajectories are suitable for tracking motion in both lateral and radial directions, as illustrated in Figure 3.

### 5.1. Scene Flow Estimation Using RGB-D Data

To generalize the concept of Dense Trajectories from 2D to 3D, we propose to make use of the 3D extension of optical flow, called scene flow. Thanks to the emergence of RGB-D cameras, numerous approaches have been proposed to estimate scene flow from depth maps, e.g., the Primal-Dual Framework for Real-Time Dense RGB-D Scene Flow (PD-Flow) algorithm [44], the Dense semi-rigid scene flow estimation [45] and the Layered RGBD scene flow estimation [46].

The scene flow Ω is linearly dependent on the depth motion field S=(u,v,w), where *w* is the range flow. It is computed by mapping S to the 3D world coordinate system as below:

(8) $$\Omega =\left(\begin{array}{ccc} \frac{Z}{f_{x}} & 0 & \frac{X}{Z}\\ 0 & \frac{Z}{f_{y}} & \frac{Y}{Z}\\ 0 & 0 & 1 \end{array}\right)S^{T},$$

where $f_{x}$ and $f_{y}$ are the camera focal lengths, and X,Y,Z are the 3D world coordinates of a specific point. On the other hand, the depth motion fields are estimated as a solution of a global variational problem, defined as:

(9) $$\begin{array}{c} \min_{S}\left\{E_{D}\left(S\right)+E_{R}\left(S\right)\right\}, \end{array}$$

where $E_{D}\left(S\right)$ is a data term defined as the combined measure of the photometric and geometric inconsistency of successive depth and intensity images and $E_{R}\left(S\right)$ is defined as a regularizer term. Multiple approximations of **S** exist based, for example, on decoupling the radial motion *w* from the lateral motion (*u*, *v*) [47,48].

We choose PD-Flow [44] to estimate a dense scene flow field from an RGB-D video stream, since it has been shown to be one of the fastest and most accurate algorithms.

### 5.2. 3D Localized Trajectories

To estimate the 3D trajectories using scene flow, we start by uniformly sampling points from the 2D image grid. In this context, we define pixel coordinates as (x,y). Similar to Wang et al. [7], we reject points belonging to homogeneous areas. Next, each of the sampled points are mapped to a standard 3D world coordinate system using the inverse of the intrinsic camera parameter matrix as described below:

(10) $$\begin{array}{c} \left(\begin{array}{c} X\\ Y\\ Z \end{array}\right)={\left(\begin{array}{ccc} \frac{(x-c_{x})D}{f_{x}} & \frac{(y-c_{y})D}{f_{y}} & D \end{array}\right)}^{T}, \end{array}$$

where $c_{x}$ and $c_{y}$ are the image plane central point coordinates, $f_{x}$ and $f_{y}$ are the respective *x* and *y* components of the focal length and *D* is the depth value. Subsequently, trajectories of the mapped 3D points are estimated using Equation (1), except that the motion field is now based on an estimated scene flow. The estimated *3D Dense Trajectories* are denoted as:

(11) $$\begin{array}{c} \left(X_{t+1},Y_{t+1},Z_{t+1}\right)=\left(X_{t},Y_{t},Z_{t}\right)+\Omega_{t}, \end{array}$$

where $\Omega_{t}$ is the scene flow field. Correspondence between estimated 3D points, with scene flow, and image pixels is derived by solving Equation (10) in terms of ${\left(x,y,D\right)}^{T}$.

The above procedure is repeated recurrently until each of the 3D trajectories reach the fixed temporal length we have set. Similar to Wang et al. [7], trajectories with sudden displacements or small overall spatial length are considered irrelevant and are removed.

In depth maps, texture information is not present. Thus, in our case, only motion descriptors are considered. Three types of descriptors are used: *3D Trajectory Shape Descriptor (3DTSD), Histogram of Scene Flow [49] (HSF)*, and *3D Motion Boundary Histogram (3DMBH)*. 3DTSD is based on the original idea of the TSD for Dense Trajectories [7]. For each trajectory, the normalized displacement vector is computed. The HSF descriptor captures the orientation and the magnitude of the local scene flow field. For a spatiotemporal volume aligned around a 3D trajectory, the orientation of the 3D displacement is calculated using the azimuth $\theta_{x,y}$ and elevation $\theta_{y,z}$ angles formed by consecutive points as:

(12) $$\theta_{xy}=\frac{\Delta Y_{t}}{\Delta X_{t}}\;\mathrm{and}\;\theta_{yz}=\frac{\Delta Z_{t}}{\Delta Y_{t}}.$$

For the histogram construction, the 4D space is quantized into a fixed number of bins. Similarly, the 3DMBH is based on the same idea as HSF. First, the derivative of the scene flow field is computed and, then, for every pair of coordinates, the orientation angle is estimated.

**Figure 4:**
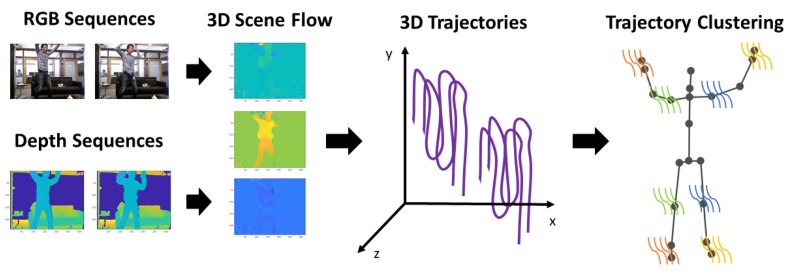
Computation steps of 3D Localized Trajectories. RGB and depth modalities are used for the estimation of the scene flow constituted of three components. Then, using the estimated scene flow, 3D Trajectories are generated. Finally, the latter are clustered around 3D body joints. A different color has been used for each cluster.

3D Trajectories are adapted to *3D Localized Trajectories* by following the procedure described in Section 4, as depicted in Figure 4. Similarly as before, we propose to enhance the discriminative power of 3D Trajectories by grouping them around 3D body joints. Hence, Equations (3)–(5) are adapted accordingly to incorporate all three dimensions of 3D trajectories $P_{3D}^{m}$ and 3D joint trajectories $Q_{3D}^{j}$. Then, during feature encoding, every histogram of joint clusters $G^{j}$ defined in Equation (6) is modified to include the descriptors used in this context, becoming:

(13) $$\begin{array}{c} H^{j}=\left[H_{3DTSD}^{j}|H_{HSF}^{j}|H_{3DMBH}^{j}\right]. \end{array}$$

### 5.3. Feature Selection for Codebook Construction

While 3D Trajectories are advantageous in capturing radial motion, they are notably noisier compared to Dense Trajectories, due to the scene flow estimation. As a result, the quality of the codebooks is degraded, unfavorably affecting the general performance of the proposed approach. This is mainly caused by the random selection of features from the training set [7] which are used to compute the final codebook. To reduce the impact of noise, we propose to select features according to the classifier *confidence* and *ambiguity* probabilistic metrics. Confidence is the classifier ability to quantify its predictions reliability, while ambiguity indicates the number of classes the classifier outputs for every prediction. The confidence C and ambiguity A metrics are defined as:

(14) $$\begin{array}{cc} C & =\underset{m\in M_{r}}{\mathrm{median}}\left(log\left(Pr\left(l_{m}=a|F^{m}\right)\right)\right), \end{array}$$

and

(15) $$\begin{array}{cc} A & =\sum_{m\notin M_{r}}\left(log\left(Pr\left(l_{m}=a|F^{m}\right)\right)\right), \end{array}$$

where $Pr(l_{m}=a|F^{m})$ is the posterior probability of label *a* given feature $F^{m}$.

Hence, the classifier is trained several times with diverse sets of random training features. In our experiments, we chose 100 sets of training features. Then, based on the computed metrics, we select the codebook which provides the highest confidence score and lowest ambiguity. If the codebook with the highest confidence is different from the one with the lowest ambiguity, we randomly select one of them. Our concept is inspired by the joint selection proposed in [11].

## 6. Experimental Evaluation

We evaluated the proposed approaches on five challenging datasets: MSR DailyActivity3D [11], Online RGB-D (ORGBD) [50], G3D Gaming [51], Watch-n-Patch [52] and KARD datasets [53]. First, a brief description of each dataset is given followed by the presentation of the experimental setups. Then, the obtained results are reported and extensively analyzed.

### 6.1. Datasets and Experimental Settings

The first dataset used for the experimental evaluation is the MSR DailyActivity 3D dataset [11]. In this dataset, 10 actors perform 16 daily activities, which in some cases involve human–object interaction. The dataset was captured by the Kinect v1 device, providing therefore RGB, depth and skeleton modalities. A distinctive characteristic of this dataset is that every actor repeats each action twice in both sitting and standing positions. For the experiments, we followed a cross-splitting protocol as in [11], where half of the subjects were used for training and the rest for testing.

The second dataset is called Online RGB-D Action (ORGBD) [50]. It can be used for both action recognition and action detection and includes seven common types of human–object interaction related to the living room environment. Three sets of video sequences were collected using a Kinect sensor. Thus, RGB, depth and skeleton modalities are available. The first set was captured in the context of action recognition in the same environment, whereas the second set was acquired for cross-environment action recognition and the third for on-line action detection. The splitting protocol requires two-fold cross-validation for the same-environment scenario, whereas, for cross-environment action recognition, training and testing sets should include different environments [50].

One challenging dataset used for the evaluation is the G3D Gaming Action Dataset [51]. This Kinect-acquired dataset can be used for both action recognition and temporal action detection. It consists of 10 subjects performing 20 gaming actions which are grouped into seven gaming scenarios: Fighting, playing golf, playing tennis, bowling, first person shooter, driving a car and miscellaneous. The first five actors were used for training and the rest were used for testing [51].

Watch-n-Patch [52] dataset, which was introduced by Cornell University, was also utilized. This dataset includes 21 types of actions (10 in an office and 11 in a kitchen) which involve interactions with 23 types of objects. Seven subjects perform 2–7 actions in each of the 458 videos. The dataset was recorded using a Kinect v2 camera. This dataset distinguishes itself by a high intra-class variability since the subjects perform different combinations of actions by ordering them differently each time. For the experiments, we used the provided splitting protocol proposed in [52], where, for every environment, almost half of the videos were used for training and the rest for testing.

The last dataset used for evaluation is called Kinect Activity Recognition Dataset (KARD) [53]. It contains 18 action classes which are performed by 10 subjects (nine males and one female). Half of the subjects were used for training and half for testing, as proposed in [53]. The dataset was captured by a Kinect device and consequently contains the three RGB-D modalities: RGB images, depth maps and 3D skeletons.

### 6.2. Implementation Details

For extracting Dense Trajectories and features from videos, we used the implementation provided by the authors in [7] (https://lear.inrialpes.fr/people/wang/dense_trajectories). The trajectory temporal length was fixed to 15 frames. The features were computed on a spatiotemporal volume of 32×32×15 aligned on the trajectory, as suggested in [7]. This volume was further divided into 2×2×3 cells, where the histograms of the descriptors were computed. In the case of 3D trajectories, we used the same parameters for the spatiotemporal volume. The number of histogram bins for the 2D trajectories was set to eight for HOG and MBH descriptors and nine for HOF descriptor, whereas for 3D trajectories case we used nine-bin histograms for every descriptor. The distance threshold for each trajectory was set to 0.02. Moreover, a linear SVM was employed for classification.

For each one of the aforementioned datasets, we report the obtained recognition accuracy using the proposed Localized Trajectories and compare it to the classical Dense Trajectories and recent state-of-the-art approaches. In the following, we denote the original dense trajectory approach [7] by Dense Trajectories. We refer to the 2D proposed approach as 2D Localized Trajectories. Similarly, the proposed 3D extension of the classical and the local Dense Trajectories are, respectively, called 3D Dense Trajectories and 3D Localized Trajectories.

The number of skeleton joints defines the number of clusters. Subsequently, in the MSR DailyActivity3D, ORGBD and G3D datasets, the skeletons are composed of 20 joints, while, in Watch-n-Patch and KARD datasets, they are, respectively, formed by 25 and 15 joints. We also empirically chose 2000 trajectories per video to construct the codebooks and 128 words per cluster and per descriptor for every dataset.

### 6.3. Performance of 2D Localized Dense Trajectories

In this subsection, an analysis of the obtained results is provided. First, we compare the performance of our approach against Dense Trajectories and other state-of-the-art methods. Later, we discuss some of the limitation of 2D Localized Trajectories.

#### 6.3.1. 2D Localized Dense Trajectories vs. Dense Trajectories

Since the aim of this work is to improve the discriminative power of classical Dense trajectories, we start by comparing our proposed 2D Localized Dense Trajectories with them. The results obtained on the five benchmarks prove the superiority of the proposed 2D Localized Trajectories. As reported in Table 1, Table 2, Table 3, Table 4 and Table 5, 2D Localized Dense Trajectories improve the accuracy by 10%, 7.7%, 3.1%, 16%, 13.8% and 0.4% on MSR DailyAvtivity3D, G3D, ORGB (same-environment settings), ORGB (cross-environment settings), Watch-n-Patch and KARD, respectively, compared to the classical Dense Trajectories [7].

**Figure 5:**
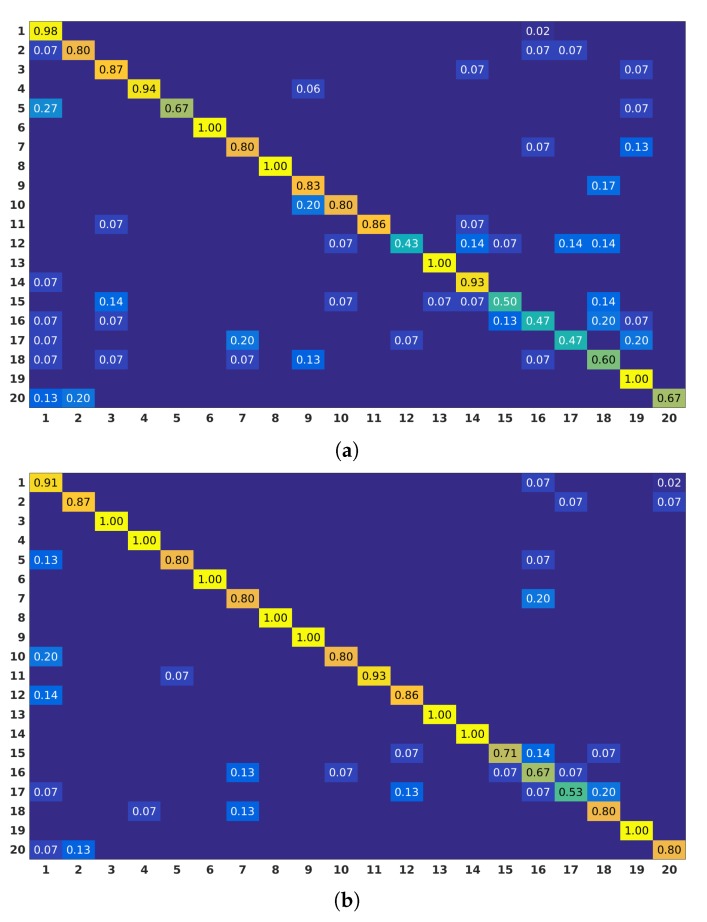
Confusion matrices obtained for Dense Trajectories (**a**) and 2D Localized Trajectories (**b**) approaches on G3D dataset. Actions list: (1) Aim and Fire Gun; (2) Clap; (3) Climb; (4) Crouch; (5) Defend; (6) Flap; (7) Golf Swing; (8) Jump; (9) Kick Left; (10) Kick Right; (11) Punch Left; (12) Punch Right; (13) Run; (14) Steer; (15) Tennis Serve; (16) Tennis Swing Backhand; (17) Tennis Swing Forehand; (18) Throw Bowling Ball; (19) Walk; and (20) Wave.

**Figure 6:**
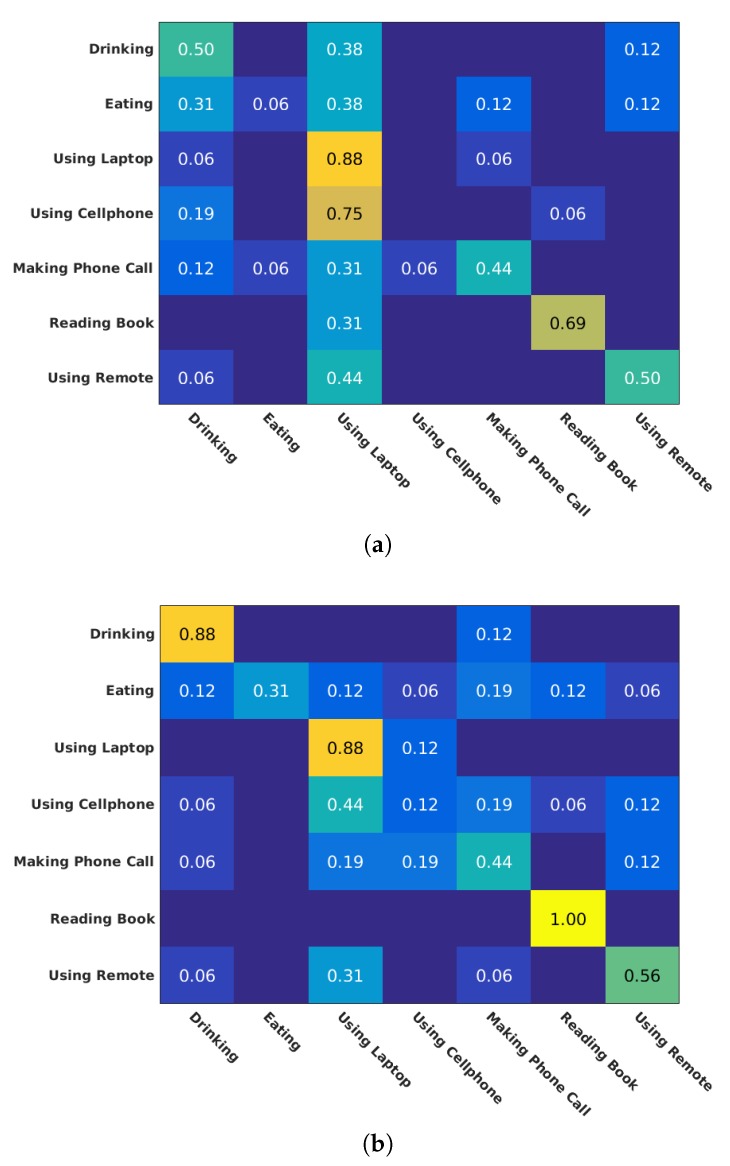
Confusion matrices obtained for Dense Trajectories (**a**) and 2D Localized Trajectories (**b**) approaches (ORGBD).

The reported results reflect the ability of 2D Localized Trajectories to distinguish actions with similar motion patterns that are performed by different body parts. This is shown in various cases when comparing confusion matrices obtained for 2D Localized Trajectories and Dense Trajectories. For instance, in the confusion matrices of G3D dataset in Figure 5, 2D Localized Trajectories boost the performance of the following action pairs: Punch Right–Punch Left and Kick Right–Kick Left. In addition, in the same dataset, the recognition accuracy of both Tennis Swing Backhand and Throwing Bowling Ball activities which include similar motion shapes is improved by 20% and 6%, respectively. Furthermore, the accuracy of Drinking and Reading Book classes in ORGBD dataset is increased by 33% and 31%, respectively (see Figure 6).

Another example of this enhancement can be the pair of actions Defend and Aim and Fire Gun in G3D dataset. The motion shapes of both action classes are similar, since both of them include arm raising. Nevertheless, the first is performed using both arms and the second by using only one arm. As we can see in Figure 5, the performance obtained for the action Defend is improved by 13% and the confusion with the action Aim and Fire Gun is reduced by 14%. In addition, in the same dataset, actions Wave and Clap have similar lateral motion and using the classical Dense Trajectories made their distinction challenging. However, with the use of 2D Localized Trajectories, motion trajectories were assigned to only one hand cluster in Wave action and to both hands in Clap action, reducing the confusion between these classes. This results in an accuracy boost of 13% in Wave class, as shown in Figure 5.

**Figure 7:**
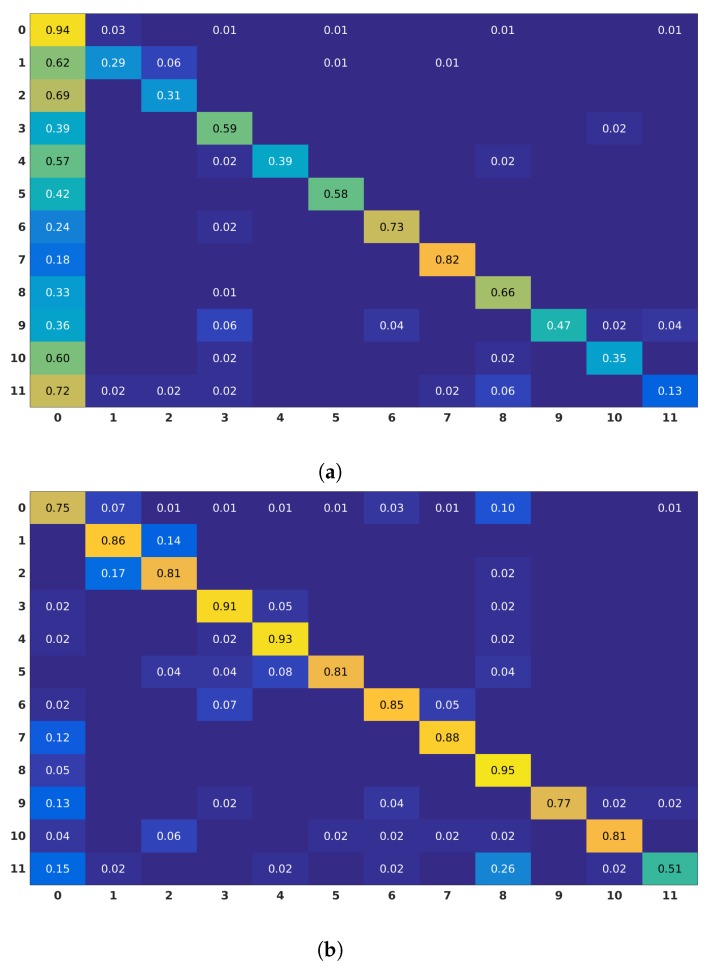
Confusion matrices obtained for Dense Trajectories (**a**) and 2D Localized Trajectories (**b**) approaches (Watch-n-Patch) in the kitchen environment. The action labels are: (0) no-action; (1) fetch-from-fridge; (2) put-back-to-fridge; (3) prepare-food; (4) microwaving; (5) fetch-from-oven; (6) pouring; (7) drinking; (8) leave-kitchen; (9) fill-kettle; (10) plug-in-kettle; and (11) move-kettle.

Moreover, in scenarios with full-body motion, such as the kitchen environment in Watch-n-Patch dataset, 2D Localized Trajectories outperform the Dense Trajectories approach, as shown in Figure 7. Clusters isolate specific motion of body parts, therefore motion patterns related to the action can be identified more effectively.

#### 6.3.2. Comparison with 3D-Based State-of-the-Art Approaches

Our 2D Localized Trajectories approach has shown competitive performance compared to 3D-based state-of-the-art approaches. In ORGBD dataset, we achieve the third best performance in the same-environment setting (Table 3). We manage to match the state-of-the-art results of Wang et al. [11] in the cross-environment settings and, at the same time, increase the mean accuracy by 16% over the Dense Trajectories.

In Watch-n-Patch dataset, the 2D Localized Trajectories improved the performance of the Dense Trajectories by 2.3% in the office environment and by 25.3% in the kitchen environment, as illustrated in Table 4. The discriminative power of our approach boosts the performance of every action class, especially in the kitchen environment, as can be observed in Figure 7. On this dataset, we only compared our work with Dense Trajectories. To the best of our knowledge, there is no work in the literature reporting offline action recognition accuracy on it, since this dataset was initially acquired for action detection.

In KARD dataset, our approach based on the 2D Localized Trajectories outperforms almost all state-of-the-art approaches, with a score of 98.2%, except JTMI, LBP and FLD [67], which reaches a slightly superior score with only 0.3% difference.

The 2D Localized Trajectories approach offers the second largest improvement on MSR DailyActivity3D dataset, by 10% compared to Dense Trajectories, as depicted in Table 1.

Finally, as reported in Table 2, our method achieves a competitive performance on the G3D dataset without the need of 3D information.

Despite the performance of 2D Localized Trajectories, it can be noted that some state-of-the-art approaches achieve better performance (e.g., [11,24,55,56,57,59,63,64,65,67]), as reported in Table 1, Table 2 and Table 3 and Table 5. We remark that most of these state-of-the-art approaches rely on 3D features [11,24,55,57,59,63,64,65,67]. Indeed, 3D descriptors are directly extracted from depth maps and/or 3D skeleton sequences. In contrast, our method computes only RGB features around the extracted 2D trajectories. The 2D information of 3D skeletons is only used to cluster the trajectories. Moreover, some of these 3D approaches (e.g., [55,57]) are even more reinforced with the use of fusion strategies. For instance, while we use only four 2D descriptors around 2D Localized Trajectories, the two aforementioned approaches [55,57] use five descriptors each. Finally, methods employing deep learning models (e.g., [56,64]) can reach higher performance, since they learn appropriate features, instead of hand-crafting them. As further investigation, it would be interesting to use a more important number of 3D features and define new strategies to fuse deeply learned and/or hand-crafted features computed around trajectories.

#### 6.3.3. Limitations of 2D Localized Dense Trajectories

**Figure 8:**
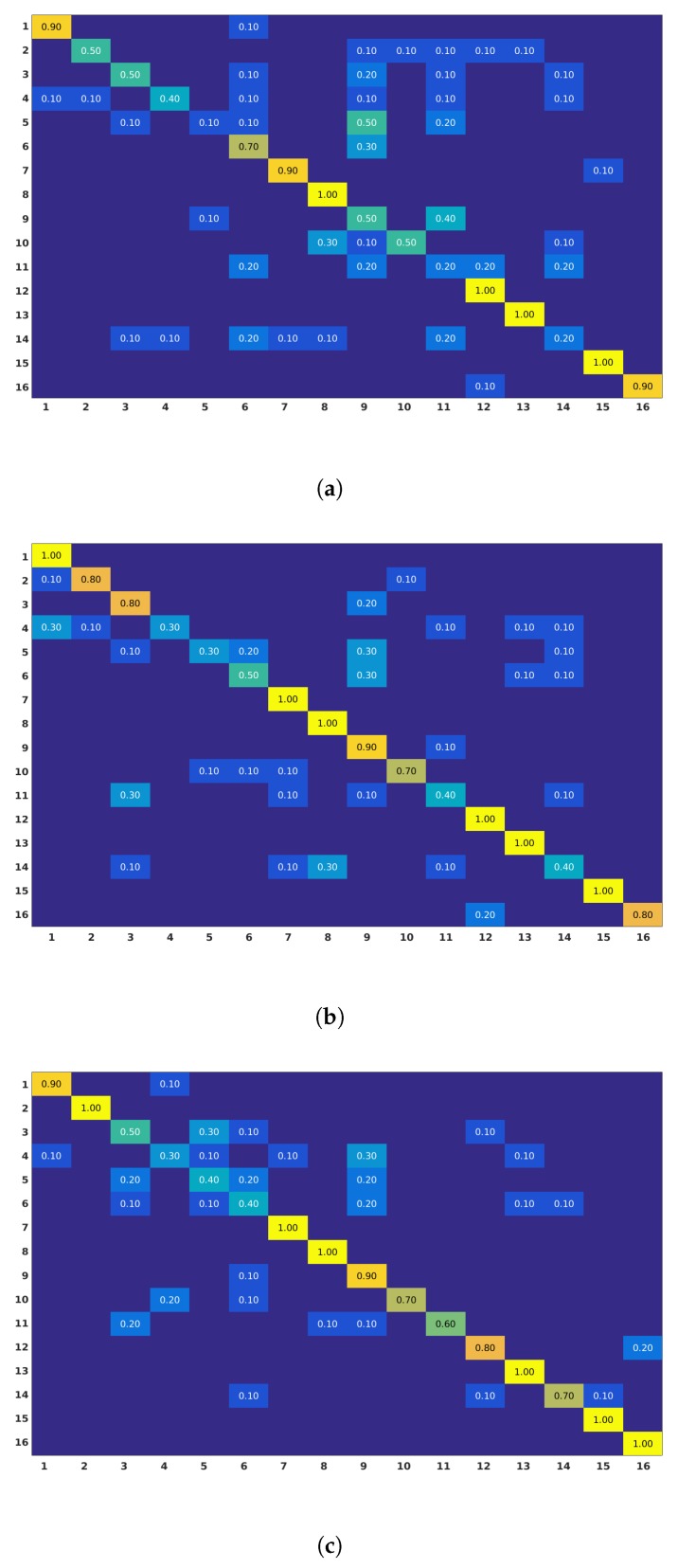
Confusion matrices obtained for (**a**) Dense Trajectories, (**b**) 2D Localized Trajectories and (**c**) 3D Localized Trajectories approaches on MSR DailyActivity 3D dataset. Actions list: (1) Drink; (2) Eat; (3) Read book; (4) Call cellphone; (5) Write on a paper; (6) Use laptop; (7) Use vacuum cleaner; (8) Cheer up; (9) Sit still; (10) Toss paper; (11) Play game; (12) Lie down on a sofa; (13) Walk; (14) Play guitar; (15) Stand up; and (16) Sit down.

Despite its strong performances, 2D Localized trajectories action representation suffers from two limitations. First, 2D Localized Trajectories approach presents low performance when the motion amount is small. This attribute is inherited from Dense Trajectories approach and is clearly depicted in action classes such as Call Cellphone in both MSR DailyActivity 3D and ORGBD, as shown in Figure 8 and Figure 6, respectively, and Write on a Paper in MSR DailyActivity 3D. Nonetheless, Sit Still class achieves adequate performance with the use of 2D Localized Trajectories, since it is an action class with almost no motion.

Second, 2D Localized Trajectories approach does not capture radial motion sufficiently. Action classes such as Playing the guitar in MSR DailyActivity3D dataset include a notable amount of radial motion and the accuracy results are consequently low, as demonstrated in Figure 8a,b. For that reason, as mentioned above, the proposed 3D Localized Trajectories presents as a good alternative to solve these two issues. Performance of the 3D Localized Trajectories are reported in the next section.

### 6.4. Performance of 3D Localized Trajectories

The proposed 3D Localized trajectories approach was evaluated on MSR DailyActivity3D and ORGBD datasets. The results reported in Table 1 show its superiority against Dense Trajectories and 2D Localized Trajectories. In fact, the accuracy of Dense Trajectories and 2D Localized Trajectories are improved by 1.9% and 11.9%, respectively. However, the reported results in Table 3 are lower than the 2D Localized Trajectories in both settings, by 2.9% and 21.4%.

The performance improvement happens mainly because of the inclusion of depth information in 3D trajectories. This helps in distinguishing actions which are performed radially with respect to the camera. The latter is particularly reflected in the confusion matrix of MSR DailyActivity 3D dataset in Figure 8, where actions such as play game and play guitar are more effectively discriminated using 3D information. The reported accuracies for the actions play game and play guitar are significantly improved. In particular, from 20% and 20% using Dense Trajectories and 40% and 40% using 2D Localized Trajectories, the accuracy climbed to 60% and 70% with the use of 3D Localized Trajectories, respectively.

Nevertheless, the results reported in Table 3 can be explained by two facts: (a) Current scene flow estimation algorithms are still very sensitive to noise in comparison to optical flow. Thus, since this dataset is slightly noisier than MSR DailyActivity3D, it is predictable to have less impressive results. However, novel approaches for a more robust estimation of scene flow are being currently investigated with the expectation of improved performance in the future. (b) 3D Localized Trajectories are more efficient than 2D ones, especially in the presence of radial motion. However, ORGBD dataset do not incorporate actions involving significant amount of radial motion. On the other hand, we can notice that some state-of-the-art methods (e.g., [24,50,55,56,57,66]) remain more accurate than the proposed 3D Localized Trajectories, as shown in Table 1 and Table 3. As explained in Section 6.3.2, the methods mentioned above make use of multiple and sophisticated 3D features directly extracted from skeleton and depth map sequences. Unlike these 3D methods, the discrimination of the features computed around the 3D trajectories is not the focus of this paper, but could be further investigated (only one 3D descriptor is used, namely HOF, while the 3D skeleton sequences are used only for the clustering of trajectories). Furthermore, our method that is based on scene flow estimation is effective especially in the presence of a high quantity of motion. On the contrary, the methods proposed in [50,66] called Ordelet and LOP4D, respectively, are effective in the presence of both high or low amount of motion, since they use local descriptors. This is confirmed by our experiments on the ORGBD dataset that incorporates actions with a low amount of motion.

These promising results highlight the potential of our first attempt to generalize Dense Trajectories to 3D and opens up new perspectives. Indeed, many components of this 3D concept can be reinforced to increase its effectiveness. For example, 3D trajectories are slightly more noisy than the Dense trajectories mainly because depth sensors introduce additional noise. This noise translated to a significant number of points belonging to the background which appeared to move radially, creating a lot of irrelevant 3D trajectories. Most importantly, the scene flow estimation is not optimal, since it relies on two different modalities which often appear to be misaligned. This fact is reflected in the performance of the 3D Trajectories (without locality), resulting in a notably lower accuracy than the Dense Trajectories, as demonstrated in Table 1. Nevertheless, the trajectory clustering around body joints is still able to remove a significant amount of noisy and irrelevant trajectories in 3D Localized Trajectories case.

### 6.5. Global BoW vs. Local BoW

To experimentally motivate the use of local BoWs, we compared the results obtained for 2D Localized trajectories using both a global BoW and a local BoWs. Hence, the experiments were conducted on the cross-environment scenario of the ORGBD dataset. The mean accuracy is notably lower compared to the 2D Localized Trajectories approach with Local BoW, reaching 53.6% vs. 59.8%. The results suggest that trajectories clustering combined with local BoWs contribute significantly to the enhancement of the local discriminative power of the overall approach. They also suggest that the local encoding is more effective, since the codebooks are constructed using features which are specific to the motion of each body part.

### 6.6. Computational Complexity

Our approach considers only a local area around each body joint. Therefore, the complexity of the proposed approach is significantly lower than the complexity of the original Dense Trajectories [7] approach. Let us denote the complexity needed to extract features around one motion trajectory by O(N), where *N* is the number of operations. While the original approach computes features around all the $K_{1}$ generated trajectories, our method conserves only $K_{2}$ trajectories within a small region around body joints (with $K_{1}\gg K_{2}$). Thus, our approach presents a lower complexity with respect to the original approach ($O\left(K_{2}N\right)\ll O\left(K_{1}N\right)$).

## 7. Conclusions

In this paper, we propose to solve two major shortcomings of the original Dense Trajectories approach using additional modalities provided by RGB-D cameras: the lack of locality information and the ineffectiveness in describing radial motion. Our contribution is two-fold. First, we enhance the discriminative power and locality-awareness of Dense Trajectories by clustering them around human body joints. This method is coupled with the local Bag-of-Words concept, strengthening further the framework. Second, we construct 3D Localized Trajectories for action recognition. For this purpose, we use: (a) scene flow instead of optical flow for the generation of the 3D Trajectories; and (b) 4D extension of the originally used spatiotemporal descriptors. The reported results show the robustness of the two proposed representations in various challenging datasets. As future work, we intend to develop an automatic way of choosing the optimal parameters. In addition, we intend to estimate more reliable and robust to noise 3D trajectories directly from point cloud data for the purposes of enhancing our current approach and extending it to view-invariant action recognition.

## Figures and Tables

**Table 1:** Mean accuracy of recognition (%) on MSR DailyActivity 3D dataset for Dense Trajectories and 2D Localized Trajectories approaches against literature.

Method	Mean Accuracy
Dynamic Temporal Warping [54]	54.0%
Local HON4D [24]	80.0%
Moving Pose [34]	73.8%
3D Trajectories [9]	72.0%
Skeleton only [11]	68.0%
Skeleton and LoP [11]	85.8%
Naive-Bayes-NN [35]	73.8%
TriViews [55]	83.8%
Skeletal Shape Trajectories [38]	70.0%
Long-Term Motion Dynamics [56]	86.9%
Spatiotemporal Multi-fusion [57]	94.1%
Dense Trajectories [7]	64.4%
3D Dense Trajectories (ours)	48.8%
2D Localized Trajectories (ours)	74.4%
3D Localized Trajectories (ours)	76.3%

**Table 2:** Mean accuracy of recognition (%) on G3D dataset for Dense Trajectories and 2D Localized Trajectories approaches against literature.

Method	Mean Accuracy
Dynamic Time Wrapping [58]	86.3%
Weighted Graph Matching [59]	89.2%
Adaptive Graph Kernels [60]	84.8%
Histogram [61]	79.5%
LPP and BoW [62]	87.5%
Spatial Graph Kernels [63]	95.7%
DL on Lie Group [64]	89.1%
Rolling Rotations [65]	88.0%
Dense Trajectories [7]	80.1%
Skeleton and LoP [11]	87.3%
2D Localized Trajectories (ours)	87.8%

**Table 3:** Mean accuracy of recognition (%) on ORGBD dataset for Dense Trajectories and 2D Localized Trajectories approaches against literature in both Same and Cross Environment Settings.

Method	Mean Accuracy	
	Same Env.	Cross Env.
Moving Pose [34]	38.4%	28.5%
Eigenjoints [35]	49.1%	35.7%
DSTIP and DCSF [26]	61.7%	21.5%
Skeleton and LoP [11]	66.0%	59.8%
Pairwise joint distance [50]	63.3%	–
Orderlet [50]	71.4%	–
Motion decomposition [66]	80.9%	–
Dense Trajectories [7]	64.3%	43.8%
2D Localized Trajectories (ours)	67.4%	59.8%
3D Localized Trajectories (ours)	64.5%	38.4%

**Table 4:** Mean accuracy of recognition (%) on Watch-n-Patch in both kitchen and office settings for Dense Trajectories and 2D Localized Trajectories approaches.

Method	Mean Accuracy
Dense Trajectories—office [7]	68.8%
Dense Trajectories—kitchen [7]	56.2%
2D Localized Trajectories—office (ours)	71.1%
2D Localized Trajectories—kitchen (ours)	81.5%

**Table 5:** Mean accuracy of recognition (%) of Dense Trajectories and 2D Localized Trajectories approaches on KARD dataset.

Method	Mean Accuracy
JTMI, LBP and FLD [67]	98.5%
JTMI and Gabor features [68]	96.0%
HOJ3D [69]	95.3%
EigenJoints [35]	96.2%
Dense Trajectories [7]	97.8%
2D Localized Trajectories (ours)	98.2%
